# Ruptured submitral aneurysm in an eight-year-old girl

**DOI:** 10.4103/0974-2069.43882

**Published:** 2008

**Authors:** Anil Kumar Singhi, Edwin Francis, Raman Krishna Kumar

**Affiliations:** Department of Pediatric Cardiology, Amrita Institute of Medical Sciences, Kochi, India

**Keywords:** Congenital submitral aneurysm, mitral regurgitation, echocardiography, CT scan

## Abstract

We present illustrative images of submitral aneurysm in a young girl. The diagnosis was established on echocardiography and the extent of the problem was defined through multi-detector computerized tomography.

## INTRODUCTION

Submitral aneurysm is a well-recognized condition of varying etiology. It is uncommon in children. It is seen as congenital anomaly, rarely presenting as complication of infective endocarditis.[1,2]

## CLINICAL SUMMARY

**Figure 1:**
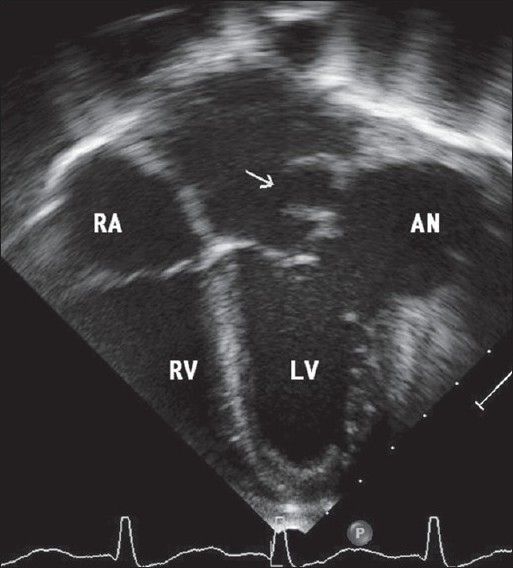
Two dimensional echocardiogram in apical four chamber view shows a large submitral aneurysm adjecant to the lateral papillary muscle of the mitral valve. It extends superiorly and is seen to rupture into left atrium (arrow)

**Figure 2:**
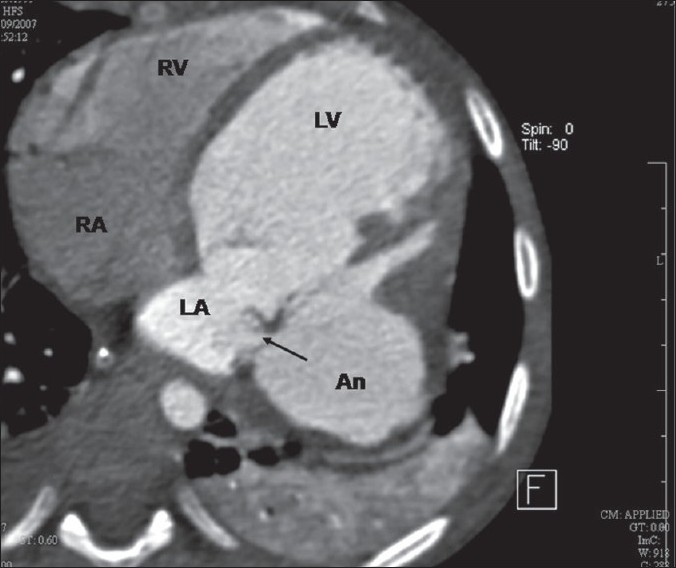
A CT scan shows the precise extent of the aneurysm and its relation to the neighboring structures. Arrow points towards the site of rupture into the left atrium

**Figure 3:**
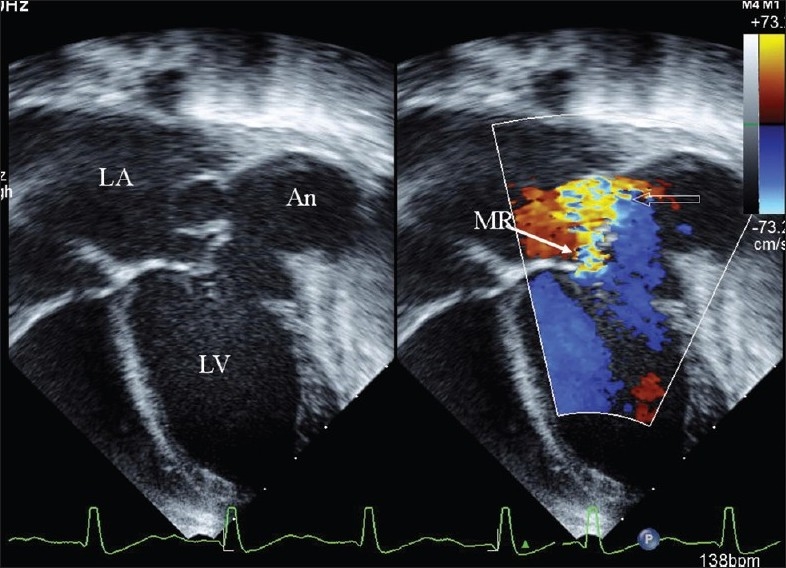
Two dimensional echo with color flow shows severe mitral regurgitation. Solid arrow points towards the regurgitant jet and the outline arrow shows the site of rupture into left atrium

An eight-year-old girl was admitted to our institution with a history of rapidly progressing respiratory distress and heart failure since three days. Her condition worsened soon after admission and she required mechanical ventilation. Her white cell count was elevated (28,000/mm^3^ ) and three blood cultures were positive for *Klebsiella Pneumoniae*. Echocardiography [Figure 1] showed a large submitral aneurysm adjacent to the lateral papillary muscle of the mitral valve tensor apparatus. It extended posteriorly and to the left of the left ventricle. The aneurysm ruptured back into the left atrium [Figures 1 and 2]. The posterior mitral leaflet was relatively immobile. A multi-detector CT scan [Figure 2] showed the extent of the aneurysm and its relationship to neighboring thoracic structures. The aneurysm distorted the mitral valve apparatus resulting in severe mitral regurgitation [Figure 3]. The child was stabilized over next few days and was operated after two weeks of antibiotics guided by the sensitivity reports. The mouth of the aneurysm was closed from within the left ventricle using a tanned autologous pericardial patch and the mitral valve was replaced using an antibiotic impregnated metallic prosthetic valve. The postoperative recovery was uneventful.

## DISCUSSION

These images are being presented to illustrate the anatomy of submitral aneurysm. Rupture of the aneurysm into the left atrium has been described and may have been responsible for the sudden worsening of the child's condition. The precise definition of all details that is necessary for planning of surgical repair can often be obtained entirely through echocardiography. However, the relationship of the aneurysm to neighboring thoracic structures requires either MRI or CT scan.
